# The Mental Health Costs of Shadow Education: The Duration of Shadow Education and Its Depressive Effects on Chinese Adolescents

**DOI:** 10.3390/bs15070885

**Published:** 2025-06-28

**Authors:** Yang Cao, Wenbin Wang

**Affiliations:** Department of Sociology, Jilin University, Changchun 130012, China; cao9508@163.com

**Keywords:** mental health, shadow education, adolescents, depressive effects

## Abstract

Shadow education refers to supplementary educational activities outside of the formal school system, typically provided by private institutions to enhance students’ academic performance. This phenomenon has become increasingly prominent worldwide, drawing significant attention from both scholars and the public. While shadow education is often associated with improved academic outcomes, its potential mental health implications for adolescents remain underexplored. Based on four large-scale surveys conducted in China from 2016 to 2022, this study examines the association between the duration of shadow education and adolescent depressive symptoms, with particular attention being paid to differences across family backgrounds and policy environments. Utilizing multilevel regression models that incorporate individual, family, and community factors, as well as inverse probability weighting regression adjustment and sensitivity analyses, this study yields three main findings. First, the relationship between shadow education duration and adolescent depressive symptoms demonstrates a U-shaped pattern: moderate engagement is associated with lower depressive symptoms, while both limited and excessive participation are linked to higher symptom levels. Second, adolescents from higher-income families are more likely to maintain shadow education participation within an optimal range and report fewer depressive symptoms. Third, following the implementation of regulatory policies on shadow education in China after 2021, the duration of shadow education among adolescents has generally shifted toward a more balanced level, accompanied by a decrease in depressive symptoms. These results underscore the need to consider the nonlinear mental health effects of shadow education in both research and policymaking. In particular, contextual factors, such as socioeconomic background and policy interventions, should be taken into account when formulating and regulating supplementary educational activities. By addressing these dimensions, policymakers can better balance the academic benefits of shadow education with its potential risks for adolescent mental health.

## 1. Introduction

The rise of shadow education has generated widespread attention and debate. In pursuit of improved academic performance, parents of adolescent students frequently enroll their children in professional tutoring institutions and extracurricular coaching classes after school (J. Luo & Chan, 2022). This pattern is especially common among parents who value academic success and can afford tutoring, yet lack the time or expertise to help their children directly. Shadow education—referring to educational activities conducted outside formal schooling—has expanded rapidly across various countries in recent years (Bray, 2024). Its growth and diffusion are driven by complex social dynamics at both the individual and group levels.

At the individual level, fierce competition for scarce educational opportunities pushes families to pursue admission to elite universities—gateways to better careers, higher incomes, and greater social standing. Access to renowned universities often requires success in rigorous examinations or contests, which families perceive as highly demanding academic benchmarks. Shadow education is thus viewed as a strategic tool to help adolescents gain an edge in these competitive processes. Parents expect shadow education to help their children outpace peers and climb the educational and social ladder (S. Li, 2023; J. Luo & Chan, 2022).

At the group level, peer and societal pressures further reinforce the prevalence of shadow education. In East Asian contexts, where academic success is highly valued, the widespread use of shadow education creates a competitive dynamic that compels other families to participate. The visibility of students gaining advantages through supplementary educational activities amplifies fears of falling behind, exacerbating competitive anxiety among remaining families (Kim et al., 2022; Pan et al., 2022; Ren et al., 2024).

As shadow education becomes an increasingly pervasive social phenomenon, its implications for educational equity and social development have prompted extensive debate. Supporters argue that shadow education fills the gaps left by classroom instruction, providing personalized and flexible learning that can boost societal human capital (Guo et al., 2020; Zheng et al., 2020). However, critics raise several concerns. First, shadow education does not consistently demonstrate clear advantages over traditional classroom instruction and may not always lead to improved academic outcomes (Guill et al., 2020; Zhao, 2015). Second, it carries the risk of inefficient resource allocation and the exacerbation of social inequalities. Substantial societal investment in shadow education may divert resources from the formal education system, potentially undermining the quality of classroom teaching. For example, skilled educators may be drawn away from formal education to the shadow education sector due to higher financial incentives, leading to a misallocation of educational talent. Furthermore, shadow education often involves higher costs and greater entry barriers than inclusive formal education, making it more accessible to privileged groups while marginalizing disadvantaged populations (Jansen et al., 2023; W. Zhang & Bray, 2018). Even in contexts with strict regulatory oversight, shadow education can exacerbate inequalities through rent-seeking behavior, influencing how educational resources are distributed (J. Li et al., 2024). Consequently, shadow education may reproduce social inequality and undermine efforts to equalize opportunity within formal schooling.

When evaluating the consequences of shadow education, it is essential to consider its potential impact on mental health. Adolescence is a pivotal stage for mental health because rapid physical, emotional, and social changes heighten exposure to stressors. At the same time, the social and emotional mechanisms for coping with stress are not yet fully developed in adolescents, rendering them more vulnerable to psychological pressures (Codjoe et al., 2024; Marthoenis & Schouler-Ocak, 2022). From a life-course perspective, many mental health problems first emerge during adolescence, with a significant proportion persisting into adulthood (Kessler et al., 2005). Adolescent mental health has, therefore, become a global concern, particularly as prevalence rates of mental health issues continue to rise worldwide. Moreover, many adolescents in low-income and middle-income countries lack timely or professional mental health care (Mudunna et al., 2025a; Twenge et al., 2021), amplifying harms to individuals and society.

Research on the determinants of adolescent mental health emphasizes the interaction between individual and environmental factors. Family migration, school victimization, and gender norms all shape adolescent mental health outcomes (Cheung, 2020; Putra et al., 2022; Xu & Ren, 2025). In the field of educational psychology, education, as both a developmental process and a social environment, plays a critical role in shaping adolescent mental health. Schooling involves not just learning content, but also ongoing interactions with peers and the broader social environment. For adolescents, education can introduce new stresses yet also offer avenues—through positive interventions—to relieve psychological pressure (Garaigordobil, 2023; X. Luo et al., 2022).

The expanding empirical literature links education and adolescent mental health, with some studies probing the causal mechanisms involved. Most research focuses on how various dimensions of the educational process relate to adolescent mental health—for example, a positive educational environment and active engagement in learning are identified as protective factors (Chen et al., 2024; Ford et al., 2021), whereas adverse school environments and poor peer relationships are recognized risk factors (Mudunna et al., 2025b). Educational expansion and the move to a knowledge economy have intensified academic pressure on adolescents, potentially contributing to rising mental health problems worldwide (Högberg, 2021). Despite ongoing debate, some studies emphasize the complexity of the relationship, calling for more rigorous examinations of causality in this area (Leurent et al., 2021; Steare et al., 2023). Advanced statistical techniques, such as randomized controlled trials and longitudinal analyses, provide stronger evidence for causal links between dimensions like academic stress, school relationships, engagement, and sense of belonging, and adolescent mental health outcomes (Singla et al., 2021; Steare et al., 2023). This complexity underscores the need for multidimensional approaches that evaluate both the academic benefits and potential psychological costs associated with shadow education.

Although shadow education can improve academic performance, its potential mental health costs warrant closer scrutiny. In the process of acquiring, producing, or maintaining something, individuals generally incur various forms of costs, most commonly in the form of money, labor, or time. However, challenges and burdens related to mental health can also constitute significant costs associated with specific behaviors and goals (David, 2011; Sahithya & Reddy, 2018). In this context, the mental health costs of shadow education refer to the psychological harm and burdens adolescents may experience while participating in shadow education in pursuit of academic success. Shadow education requires considerable time and financial investment from both adolescents and their families, and can also impact adolescents’ mental health status. As a significant form of education, shadow education is inherently connected to academic performance and directly influences the degree of academic stress experienced by students. Moreover, because shadow education operates outside the standardized boundaries of formal schooling, it often overlaps with leisure time, reducing opportunities for rest and relaxation, which may intensify psychological pressures (Kuan, 2018; D. Wang et al., 2022). In short, tutoring can simultaneously aggravate and alleviate stress, giving it a complex role in adolescent mental health. Consequently, participation in shadow education continuously shapes adolescent mental health, and students may incur lasting psychological costs in their pursuit of academic success.

Nevertheless, the complex causal relationship between shadow education and adolescent mental health has not been fully established. Accordingly, the discussion of mental health costs in this study is grounded in the association between shadow education and adolescent mental health, rather than definitive causality. Determining how much shadow education actually costs adolescents psychologically will require studies that use rigorous causal inference methods.

Despite increasing attention to the academic outcomes of shadow education, its effects on adolescents’ mental health remain insufficiently explored. Most existing studies focus on whether shadow education improves academic achievement or which groups of students benefit most academically (Bray, 2014; Hajar & Karakus, 2022). Only limited research has investigated the mental health implications, leaving significant gaps in understanding the conditions under which shadow education affects psychological well-being. Some scholars argue that shadow education not only enhances academic performance, but also serves as a coping strategy for adolescents and their parents to manage academic pressure and alleviate anxiety (Sun et al., 2020). Conversely, critics highlight that, as an activity situated outside the official curriculum, shadow education inevitably encroaches upon adolescents’ leisure time, increases academic burdens, and introduces additional sources of psychological stress (Hu & Mu, 2020).

Reaching a consensus on how shadow education affects adolescent mental health remains a complex issue. Further research is needed to clarify the conditions under which shadow education produces positive or negative mental health outcomes. This requires a nuanced examination of factors such as the duration of shadow education and the broader context in which it occurs, including family dynamics, educational policies, and societal environment.

China provides a compelling context for examining shadow education amid shifting regulatory landscapes. The development of shadow education practices varies considerably across countries, shaped by differing policy approaches and cultural priorities. Some nations, such as Canada, the United Kingdom, Singapore, Nigeria, and South Africa, have adopted laissez-faire or supportive policies, thereby fostering the growth of shadow education. In contrast, countries like Vietnam, South Korea, Myanmar, and Lithuania have imposed strict supervision or outright bans, actively regulating or restricting shadow education practices (Silova et al., 2006, p. 14). In China, shadow education has undergone significant transformations, driven by cultural emphasis on academic success and evolving government policies. Before 2018, shadow education in China largely prospered under a laissez-faire approach. By 2020, the sector encompassed over 4 million training institutions and employed more than 11 million individuals. Shadow education not only operated alongside the formal education system, but, in some respects, surpassed it, raising concerns regarding educational equity and academic burden (W. Zhang, 2023, p. 61). In response, the Chinese government introduced a series of regulatory interventions, culminating in the implementation of the “Double Reduction” Policy (“Shuang Jian Zhengce”) in 2021.

The “Double Reduction” Policy is a comprehensive educational reform launched by the Chinese central government, targeting adolescents at the compulsory education stage. Its title reflects two primary objectives, reducing both the excessive homework burden assigned by schools and the heavy reliance on private after-school tutoring. The policy emerged as a response to mounting public concerns over escalating academic pressure and educational inequities exacerbated by the expansion of shadow education. The rationale behind the “Double Reduction” Policy acknowledges that the flourishing private tutoring industry had become a major source of stress for families and students. To address these challenges, the policy introduced a series of concrete measures. First, it strictly regulates both the quantity and difficulty of school-assigned homework, such as prohibiting written homework for lower primary students and setting clear time limits for other grades. Second, it targets the for-profit tutoring industry by banning the provision of tutoring in core subjects on weekends, holidays, and school breaks, and requires all academic tutoring organizations serving compulsory education students to register as non-profit entities. Additionally, the policy enforces strict financial regulations, including bans on public listings and foreign investment in tutoring companies, to curb the commodification of education.

The effects of these regulatory reforms have been significant, as evidenced by a sharp decline in the number of officially registered shadow education institutions and employees, effectively curbing the sector’s rapid expansion. Nevertheless, shadow education has not disappeared entirely from Chinese society. Instead, educational providers have adapted by rebranding services and modifying instructional methods, effectively bypassing some of the policy restrictions while continuing to meet persistent demand. Empirical studies from China indicate that, following the introduction of the Double Reduction Policy, the time and financial investment adolescents devote to shadow education have generally declined. However, the policy’s impact varies across socioeconomic groups, with middle-income families experiencing the most significant changes, while high-income and low-income groups remain less affected (Pang et al., 2025).

These dynamics make China a compelling case for examining the mental health implications of shadow education. On the one hand, the phenomenon is deeply embedded in China’s social and cultural fabric, reflecting the high value placed on education in East Asian societies and intense competition for educational resources. Shadow education, therefore, shapes not only academic outcomes, but also the mental well-being of Chinese adolescents. On the other hand, the mental health consequences of shadow education are closely linked to social context. The evolving regulatory environment in China provides a unique opportunity to investigate how changes in policy influence the relationship between shadow education and adolescent mental health. Differences in family background and parental decisions, shaped by shifting regulations, are likely to modulate shadow education’s mental-health effects.

To better understand the mental health consequences of shadow education, this study utilizes four large-scale survey datasets from China spanning 2016–2022. Within this framework, the duration of shadow education is examined, with depressive symptoms serving as indicators of mental health. The study analyzes how shadow education duration is associated with adolescents’ depressive symptoms, as well as the influence of social and contextual factors across different family backgrounds and regulatory environments.

## 2. Literature Review and Hypothesis Development

### 2.1. Stress Relief Mechanism or Source of Pressure: The Relationship Between Shadow Education and Mental Health

Shadow education can function both as a mechanism for relieving stress and as a source of pressure for adolescents, thereby influencing their mental health in complex and multifaceted ways.

As a stress-relief mechanism, shadow education may improve adolescents’ psychological well-being through two primary pathways. First, it provides access to broader and more diverse learning resources, which can enhance academic performance. Improved performance may foster adolescents’ sense of achievement and self-efficacy, enabling them to navigate competitive educational environments with greater confidence. These academic gains may lessen worries about forthcoming challenges and thus support better mental health. Second, shadow education participation may offer psychological reassurance to adolescents and their parents, especially in highly competitive settings. Although shadow education is not invariably pleasant or effortless, in highly competitive settings, it often provides a measure of reassurance. Conversely, non-participation can increase anxiety by signaling a risk of falling behind, even if this risk does not materialize. Once involved, the act of participating itself can provide a sense of psychological security, particularly by alleviating pressures associated with peer comparison (Sun et al., 2020; Zheng et al., 2020).

In contrast, shadow education may also serve as a significant source of psychological pressure through two main pathways. First, increased hours of shadow education reduce adolescents’ sleep and leisure time. With the limited time available outside formal schooling, intensive shadow education often encroaches on crucial rest and recreation, impairing physical and emotional well-being. This reduction in both the quantity and quality of sleep and leisure increases the risk of negative emotions and psychological distress, including anxiety, depression, and irritability (Ahn & Yoo, 2022; Moreno & Jurado, 2024; Yao & Chen, 2023). Second, shadow education can amplify the burden of social expectations. Parents often view it as an investment in their children’s academic success, anticipating concrete improvements in performance. This perspective creates strong familial and social pressure for adolescents, compelling them to participate regardless of their personal interest or intrinsic motivation. When shadow education fails to deliver the expected academic improvements, discrepancies between adolescents’ actual outcomes and parental expectations may exacerbate psychological distress (Pearlin, 1989; Xu et al., 2024).

Because of these dual effects, empirical work continues to report a complex link between shadow education and mental health. Some studies emphasize its potential to relieve stress, reporting positive outcomes—for example, research from China suggests that adolescents who participate in shadow education may exhibit better mental health (Sun et al., 2020). Conversely, a substantial body of the literature underscores the negative consequences, linking extracurricular tutoring to an increased likelihood of depressive symptoms and poorer mental health among adolescents (Hu & Mu, 2020; Kim et al., 2022). These findings suggest that the mental health impact of shadow education is highly context-dependent, and further investigation is needed to clarify the underlying mechanisms.

To address these complexities, the present analysis employs two complementary strategies. First, we investigate the differential effects of varying durations of shadow education, alongside the familial and regulatory factors that shape these patterns. Understanding how duration influences mental health outcomes is critical for distinguishing beneficial effects from detrimental ones. Second, we focus on depression as a measurable indicator of mental health. Although mental health encompasses a broad and sometimes ambiguous array of factors, depression represents a prevalent and concrete symptom that effectively reflects the mental health status of adolescents.

### 2.2. Nonlinear Effects of Shadow Education Duration on Depression

The ongoing debate regarding shadow education may stem from insufficient attention to participation duration as a critical factor. Most existing studies assess shadow education participation using a binary indicator—simply distinguishing between “involvement” and “non-involvement”—which lacks nuance and precision. However, shadow education involves a diverse array of formats, delivery modes, and intensities, necessitating more detailed measurement approaches (Bray, 2023; Hajar & Karakus, 2024). For example, adolescents may participate in shadow education through one-on-one tutoring, small group sessions, large classes, or online courses. Participation may be related to regular school subjects or independent enrichment, occur after school or on weekends, and be provided by either professionally trained or untrained tutors. Thus, the “how” of participation—rather than simply “whether” adolescents engage in shadow education—carries significant implications for academic and mental health outcomes. This necessitates a closer examination of key characteristics of shadow education.

This study focuses on the duration of shadow education as a critical factor, aiming to address limitations in existing measurement approaches and provide a more detailed depiction of the intensity, demands, and potential burdens associated with adolescents’ involvement. For instance, a teenager who participates for one hour per week and another who engages for forty hours are often classified similarly in research, despite stark differences in their experiences and outcomes. Such differences are likely to produce distinct effects on academic outcomes and mental health. Therefore, it is insufficient to simply account for participation status; duration must also be closely examined to capture the nuanced effects of shadow education.

The relationship between shadow education duration and its effects is not necessarily linear; instead, it often follows a nonlinear trajectory. Some studies have found that the association between shadow education duration and academic achievement is characterized by an inverted U-shape: moderate engagement improves academic performance, but excessive participation yields diminishing returns and may even lead to negative effects. (Hof, 2014). Although the precise location of this turning point may vary depending on individual and contextual factors, the general pattern includes three stages: insufficient education, appropriate education, and excessive education.

Insufficient education refers to little or no participation in shadow education, resulting in missed opportunities for academic enhancement. In this stage, increasing shadow education typically leads to academic improvement. Appropriate education describes engagement at a moderate, “optimal” level, where participation yields maximum academic benefits without imposing excessive psychological burdens. However, when duration exceeds an individual’s capacity or the optimal threshold, excessive education occurs. In this phase, additional hours not only fail to improve academic outcomes, but may also increase stress, reduce intrinsic motivation, and displace valuable leisure time (Cheo & Quah, 2005). For students with strong academic abilities and high self-direction, excessive shadow education may be particularly counterproductive, as their need for additional instruction diminishes and their autonomy is undermined (L. C. Wang, 2015).

When examining the impact of shadow education duration on adolescent mental health, it is crucial to consider whether this relationship is linear. Increasing evidence from studies on adolescent behaviors and health outcomes suggests that such relationships are often nonlinear, with moderate levels frequently yielding the most favorable outcomes. The “moderate is best” principle aligns with theoretical frameworks such as optimal stress theory and moderation theory. These frameworks emphasize that both deficiency and excess can lead to negative consequences, while balanced involvement produces optimal results.

For example, the Yerkes–Dodson Law posits a nonlinear, inverted U-shaped relationship between motivational intensity and performance (Teigen, 1994). When motivation or stress is too low, individuals may lack sufficient drive, resulting in sluggish or poor performance. Conversely, excessive motivation or stress increases anxiety, tension, and the likelihood of errors, thus impairing overall performance. Only under moderate levels of motivation and stress can individuals achieve their best outcomes, as this balance enhances focus, effort, and emotional regulation. Applied to shadow education, this theory suggests that moderate participation enables adolescents to experience sufficient academic challenge while maintaining their cognitive and emotional well-being. For instance, moderate participation may promote goal-setting, discipline, and self-efficacy—all factors that contribute positively to mental health. By contrast, minimal engagement may fail to provide adequate cognitive stimulation, leading to underachievement and feelings of inadequacy, while excessive involvement can result in heightened anxiety, fatigue, and burnout, which undermine mental health.

Another relevant theoretical framework is the Goldilocks Principle, which underscores the idea that optimal outcomes occur when a balance is struck—not too little and not too much (Balas-Timar & Lile, 2015; Straker et al., 2018). This principle complements the Yerkes–Dodson Law by emphasizing the importance of achieving “just-right” levels of engagement, where academic challenges are stimulating without compromising psychological well-being. Specifically, moderate shadow education participation allows adolescents to develop critical academic skills while preserving time for essential activities like leisure, social interactions, and rest, all of which are crucial for maintaining mental health. This principle has also been validated in related fields of adolescent mental health, such as studies on sleep and physical activity, which similarly demonstrate U-shaped relationships (Qu et al., 2025).

Compared to a linear model, which implies a constant increase or decrease in depression with shadow education duration, a U-shaped model better reflects the dynamics of adolescent mental health. Specifically, both very low and very high levels of engagement are problematic, whereas moderate involvement is optimal. The linear model fails to capture this “optimal zone,” nor does it account for the reversal of effects at the extremes, thus risking oversimplification and potentially misleading policy or parental decisions. By contrast, the U-shaped curve identifies a threshold or “critical zone” for shadow education duration, emphasizing that excessive or insufficient involvement can have detrimental consequences for adolescent mental health. This theoretical framework aligns with empirical findings and offers practical implications for guiding educational policies and parenting strategies.

Accordingly, the classification of insufficient, appropriate, and excessive education is a useful heuristic for analyzing the relationship between shadow education and adolescent mental health. When the duration remains within a certain threshold, it meets adolescents’ learning needs while alleviating mental stress, thereby improving their competitive advantage and self-efficacy while reducing anxiety and depression. In this scenario, adolescents benefit both academically and psychologically, as the balance between academic challenge and mental well-being is maintained. Conversely, those who opt out or participate minimally may experience educational insufficiency, with lower academic and mental health outcomes compared to their peers in the “adequacy” range. However, once the duration exceeds this critical threshold, it may overwhelm adolescents’ coping capacity. Prolonged engagement can further encroach on sleep and leisure time, impose additional financial and learning burdens, and serve as a new source of psychological stress and depressive symptoms (Yao & Chen, 2023).

Therefore, a U-shaped model more accurately characterizes the relationship: both too little and too much shadow education are linked to poorer outcomes, while a moderate amount is most beneficial. The linear model’s failure to capture this optimal range risks oversimplifying the relationship and producing misguided recommendations.

Given these considerations, the relationship between shadow education duration and adolescent mental health can be classified into three states: insufficient education, appropriate education, and excessive education. However, it is theoretically challenging to precisely define the thresholds separating these categories, as they depend on individual abilities, personality traits, and contextual factors, and no consensus has yet emerged in the literature (Hof, 2014). Nevertheless, identifying an approximate threshold remains necessary from both theoretical and practical perspectives. Establishing such a threshold enables researchers to pinpoint the bottom of the U-shaped curve and confirm the presence of nonlinear trends, while also providing policymakers and parents with a more reliable foundation for decision-making in educational planning.

Drawing on empirical evidence from Chinese adolescents’ after-school study patterns, this study tentatively defines the upper threshold for “adequate” shadow education participation at approximately 21 h per week, or about 3 h per day. This estimation is based on two key considerations: one practical and the other theoretical.

First, on a practical level, a daily average of three hours of shadow education is both common and meaningful. Within the context of Chinese society, weekdays typically see less shadow education participation, while weekends often involve higher engagement levels. As a result, shadow education participation over the course of a week tends to approximate a daily average of three hours. Similarly, international empirical studies indicate that after-school study durations for adolescents frequently fall within a range from three to four hours per day (Galloway et al., 2013; Theobald, 2025). This suggests that the benchmark of 3 h per day, or 21 h per week, is grounded in practical realities and provides insights that are applicable across different contexts. Moreover, this threshold offers meaningful guidance for understanding adolescent mental health across diverse cultural settings.

Second, from a theoretical perspective, the proposed threshold is consistent with frameworks emphasizing the balance between academic demands and psychological resources among adolescents. For instance, the 24-hour Activity Cycle framework provides a structured guideline for balancing different daily activities, including formal schooling, sleep, physical activities, and leisure. According to existing research, adolescents should ideally have 8–9 h of sleep, 60–90 min of napping, and roughly 1 h of outdoor physical activity each day, alongside approximately 7 h of formal schooling and 2 h of mealtime (Kumar & Chowdhury, 2021; Qu et al., 2025). This leaves adolescents with approximately 5 discretionary hours per day. Considering the need for leisure and social interactions, three hours of shadow education fits within this discretionary time without overly compromising other essential activities.

However, the 21-h threshold should not be regarded as a rigid or universal benchmark. While it offers initial insights into the balance between shadow education and adolescent mental health, further research is needed to assess the stability of this threshold across subsamples, such as by gender, age, and geographical location. For example, cultural expectations and socioeconomic factors may influence adolescents’ engagement in shadow education, potentially shifting the optimal threshold. Similarly, individual differences in coping capacity or academic resilience could result in variations in the inflection point. To address these possibilities, future studies should employ stratified analyses to evaluate whether this threshold is consistent across diverse demographic or contextual factors.

Nonetheless, the classification into insufficient, adequate, and excessive shadow education remains a valuable heuristic for identifying nonlinear trends. This is clearly not a precise dividing point, nor a strict policy benchmark. It is important to emphasize that this threshold serves as a provisional guideline rather than a definitive metric for policymaking or parental decision-making. Rather than proposing this threshold as final, it represents a starting point for further theoretical refinement and empirical validation. Accurate policy recommendations would require more rigorous causal inference strategies and a broader consideration of individual characteristics and cultural contexts.

Based on this, Hypothesis H1 can be proposed:

**H1.**  *The relationship between shadow education duration and adolescent depression follows a U-shaped curve.*

### 2.3. Social Contextual Features: The Influence of Family and Policy

If a critical threshold exists in the mental health costs of shadow education duration, a key question arises: which groups are best positioned to recognize and utilize this threshold to maximize academic performance while minimizing psychological burdens? The theory of social determinants of health posits that, beyond the direct causes of illness, individuals’ social status and access to resources significantly influence their overall social environment throughout their life cycle—from birth and development to work and aging—ultimately affecting their health outcomes (Nutbeam & Lloyd, 2021).

Shadow education, as a form of educational investment at the family level, is deeply influenced by the socioeconomic characteristics of adolescents’ families. Families vary in their access to social resources, such as wealth, power, and social capital, which not only influence adolescents’ participation in shadow education, but also affect the mental health consequences associated with such participation. Among these resources, family income is particularly critical, as it determines access to shadow education while shaping the psychological outcomes of involvement. Families with higher income occupy an advantageous position in the distribution of social resources, enabling them to actively support their adolescent members’ involvement in shadow education. Because shadow education can reproduce social advantage, it allows for wealthy families to convert economic resources into additional educational gains. Therefore, adolescents from higher-income families are more likely to engage in shadow education and benefit from it both academically and mentally (Bray, 2023; Song & Zhou, 2019).

Two mechanisms explain why higher-income families manage shadow education’s mental health costs more effectively: First, adolescents from higher-income families can access more effective strategies—such as improved sleep environments and a wider range of recreational activities—to offset the psychological stress generated by shadow education. Second, these families are better positioned to select appropriate durations of engagement, thereby avoiding the risks of over-participation or under-participation. Specifically, higher-income households are more likely to translate shadow education participation into actual academic progress, while lower-income families may face greater pressure to over-invest in shadow education, exacerbating mental stress. Conversely, adolescents from higher-income families tend to adopt a more balanced perspective, reducing the likelihood of over-participation and the attendant mental-health costs (Gao & Xue, 2021; Z. Li & Qiu, 2018).

As a result, adolescent from higher-income families not only manage the mental-health costs of shadow education more effectively, but also enjoy a broader range of stress-relief options. Additionally, they are more likely to approach shadow education rationally, selecting programs of reasonable duration that impose lower levels of mental stress. These factors collectively reduce the negative impact of shadow education on their levels of depression. Based on this reasoning, Hypotheses H2 can be proposed:

**H2.**  *As family income increases, adolescents from higher-income families are more likely to experience lower levels of depression.*

Furthermore, it is essential to consider the implications of the Chinese government’s 2021 “Double Reduction” policy for shadow education. The introduction of this policy has significantly curbed the expansion of shadow education and formally discouraged indiscriminate participation by minors. As a result, the legitimacy and social foundation of shadow education have weakened, prompting families to reassess its effectiveness rather than yielding to peer pressure to enroll their adolescents.

In this new context, several shifts may alter the relationship between shadow education and adolescent depressive symptoms. First, any influence of shadow education on academic performance and mental health is likely to be amplified. As regulatory constraints increase, remaining shadow education providers are incentivized to improve their educational quality and align services with national priorities such as equity and holistic development (Yang et al., 2024). Many institutions are shifting from a narrow focus on exam preparation for elite students to a broader emphasis on holistic and inclusive development, thereby potentially reducing educational inequality and offering more practical support to a wider range of adolescents. Properly designed shadow education programs under this framework could deliver meaningful gains in both academic performance and mental health.

Second, rising costs and regulatory risks are expected to prompt more cautious participation among consumers who were previously uncritical. As shadow education institutions focused on exam preparation come under closer scrutiny, some may be forced to operate covertly, increasing costs and risks for participants (Liu & Bray, 2022). This environment may temper irrational enthusiasm and promote more measured, deliberate decision-making regarding participation.

Third, peer and societal pressures to join shadow education are likely to decline. The “Double Reduction” policy clearly signals state disapproval of excessive shadow education, and, combined with declining participation rates, reduces competitive pressures on families and adolescents. This is particularly beneficial for those previously compelled to participate due to social competition within their peer groups.

In summary, the “Double Reduction” policy is expected to steer parents and adolescents toward more rational and effective choices regarding shadow education, thereby reducing its negative mental health impact while enhancing potential benefits.

However, it is critical to acknowledge the potential interference from external factors, particularly the COVID-19 pandemic, which coincided with the implementation of the “Double Reduction” policy. The pandemic, which began in early 2020 and persisted until early 2023 in China, profoundly affected both shadow education and adolescent mental health. Formal education was frequently disrupted, prompting many parents to turn to online shadow education as a substitute, especially among families with lower parental education levels who lacked the capacity to provide academic support at home (Du, 2024). At the same time, numerous studies have documented a decline in adolescent mental health during the pandemic, driven by increased psychological stress and the prolonged duration of shadow education (Sayed et al., 2024).

Thus, the effects of the “Double Reduction” policy and COVID-19 may be distinguished, as their directions and timing do not always align. During the pandemic, participation in shadow education likely increased and adolescent mental health deteriorated—outcomes contrary to the aims of the “Double Reduction” policy. The policy’s implementation in July 2021 occurred in the latter part of the pandemic, making trends in adolescent depression and shadow education participation after 2021 critical for distinguishing the policy’s effects from those of COVID-19. If mental health and shadow education participation trend upward after 2021, the pandemic’s effects may be dominant; conversely, a notable decline in depression and participation would suggest the beneficial influence of the “Double Reduction” policy.

Given its clear focus and rapid nationwide rollout, the “Double Reduction” policy is likely to exert an independent, positive influence on shadow education and adolescent mental health by encouraging more judicious participation and reducing depressive symptoms. Based on this reasoning, Hypothesis H3 can be proposed.

**H3.**  *After 2021, adolescents are more likely to experience lower levels of depression.*

## 3. Methods

### 3.1. Data Source

The data for this study are drawn from the China Family Panel Study (CFPS), a nationally representative and comprehensive social survey designed to collect data at the individual, family, and community levels. The CFPS tracks changes in China’s social, economic, demographic, educational, and health landscape. Initiated by a research team at Peking University in 2010, the CFPS conducts surveys from every one to two years, with the latest publicly available wave fielded in 2022.

The CFPS sample covers 25 provinces in China, representing approximately 95% of the total population. The survey employs a three-stage, implicitly stratified, probability sampling design. The primary sampling units (PSUs) are administrative districts or counties, the secondary sampling units (SSUs) are administrative villages or neighborhood committees, and the tertiary sampling units (TSUs) are households. Official administrative-division data guide the first two stages, and a map address method is used to construct the household frame in the third stage. Trained interviewers then conduct face-to-face computer-assisted interviews in respondents’ homes.

This study draws on data from the 2016, 2018, 2020, and 2022 CFPS waves for three reasons. First, a longer observation window facilitates a more comprehensive analysis of trends in adolescent depression and shadow education. Second, the selected period encompasses the year 2021, a critical point when the Chinese government implemented substantial regulatory policies on shadow education, enabling the assessment of these policies’ impacts. Third, adolescent subsamples are often limited in large-scale social surveys. By pooling data from these years—each containing the core variables of interest—substantially increases the adolescent sample size and enhances statistical power.

The CFPS project is a human subjects research initiative. To ensure participant protection, the research team regularly submits ethical review or ongoing review applications to the Biomedical Ethics Committee of Peking University. Data collection was conducted only after receiving approval from the ethics review.

For the purposes of data analysis, the adolescent sample was restricted to those aged from 10 to 15, for two main reasons. First, individuals in this age range experience rapid physical and psychological development, along with a certain degree of cognitive and emotional maturity. Second, this age group is at a pivotal stage of academic development, faces considerable academic pressure, and represents a primary clientele for shadow education. After rigorous data cleaning, the final analytic sample comprised 7739 adolescents.

Out of the initial 8773 adolescent observations, 1034 cases were excluded due to missing information on variables relevant to this study. Given the relatively low percentage of missing data (11.79%), listwise deletion was employed. To assess potential bias from missing data, a three-level binary logistic regression model was used to predict missingness. The results indicate that lower body weight was associated with a higher likelihood of being missing, while all other variables—including key explanatory variables such as shadow education duration and family income—were not significantly associated with missingness. Thus, there is no empirical evidence that missing data would substantially bias the study’s main results.

### 3.2. Variables

The dependent variable in this study is the level of depression, measured with the Center for Epidemiologic Studies Depression Scale (CES-D) developed by Radloff (Radloff, 1977). Respondents are asked to self-report the frequency of depressive symptoms experienced over the past week, with four response options: Rarely, Sometimes, Often, and Most of the time. The CES-D comprises 20 items, including statements such as “I worry about small things,” “I feel lonely,” “I think others dislike me,” and “I find it hard to do anything.” Responses are summed to generate a continuous total depression score, with higher scores indicating more severe depressive symptoms and poorer mental health.

There are numerous instruments available for measuring depression, such as the Beck Depression Inventory (BDI), Positive and Negative Affect Schedule (PANAS), Patient Health Questionnaire-9 (PHQ-9), and Patient-Reported Outcomes Measurement Information System (PROMIS), among others (Amtmann et al., 2014; Jiang et al., 2019). The selection of the CES-D for this study is based on two considerations. First, the CES-D is widely used across diverse research populations and topics, demonstrating high internal consistency and test–retest reliability, with Cronbach’s alpha coefficients typically ranging from 0.85 to 0.90 (Miller et al., 2008). Its discriminative validity for mental health has been consistently supported across different studies and regions, including China (M. Wang et al., 2013). Second, although the CES-D is not intended for clinical diagnosis, it is highly sensitive to subclinical depressive symptoms in community and non-clinical populations, making it particularly suitable for large-scale epidemiological surveys. Given the objectives of this study, the CES-D is able to capture a wide spectrum of depressive symptoms among adolescents and provides a stable reference for mental health comparisons across different years.

The independent variables include three components. The first independent variable is the duration of shadow education. This is calculated by summing the time respondents spend each week participating in five types of shadow education activities, including tutoring, competition preparation, and cognitive development. The total time spent on shadow education each week is represented as a continuous variable.

The second independent variable is family income, which is measured by the per capita annual income of respondents’ households. Given that income may be skewed, a logarithmic transformation is applied to adjust for this distribution, resulting in a continuous variable that represents family income.

The third independent variable is the survey year. The original time points are adjusted to reflect three periods: before 2018, 2020, and 2022. This adjustment serves two purposes: first, to capture the potential impact of changes in China’s shadow education regulatory policies around 2021 on shadow education and mental health; and second, to account for the global pandemic in 2020, which may have caused abrupt changes in mental health due to external environmental factors, necessitating a distinction in the analysis.

Additionally, control variables were established across three levels: individual, family, and community. This approach is based on two primary considerations. First, the extant literature on the determinants of shadow education and mental health consistently emphasizes the importance of both individual-level and group-level factors, necessitating their simultaneous inclusion in the analysis. Second, the multi-level sampling design of the CFPS dataset enables the use of multi-level regression models to robustly control for variables across individual, family, and community levels.

Specifically, individual-level control variables include age, gender, years of education, height, weight, physical health, academic performance, incidence of absenteeism, class leadership position, and internet usage. Family-level variables include whether the adolescent is a left-behind child and the frequency of arguments with parents, both of which capture family environment influences. Community-level variables include the proportion of left-behind children in the community and the average academic performance of children in the community, which primarily control for the effects of community environment and peer pressure. To mitigate potential multicollinearity, all family-level and community-level control variables were mean-centered prior to analysis.

**Figure 1:**
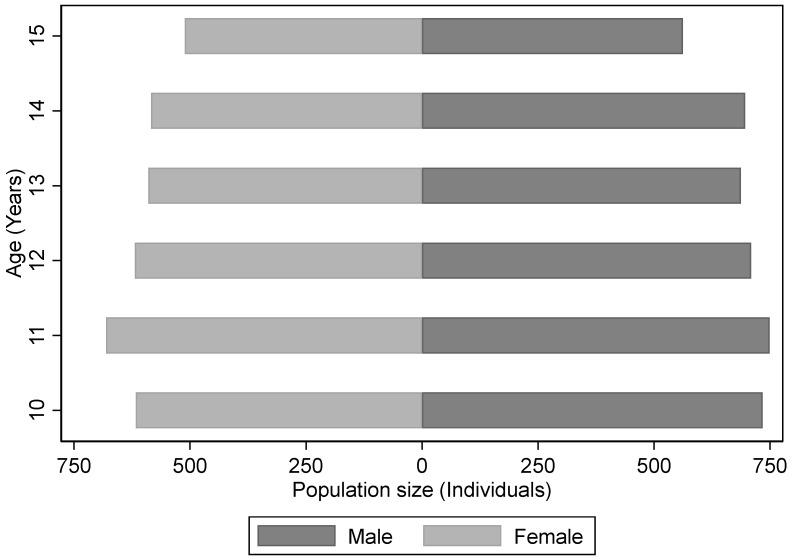
Age and gender pyramid of the analytical sample.

To present the basic demographic characteristics of the analytical sample, a population pyramid depicting the distribution of age and gender was plotted, as shown in Figure 1.

Figure 1 shows that, among adolescents aged from 10 to 15, the gender distribution remains generally balanced—although males outnumber females at each age, a pattern consistent with overall population census data in China. In terms of age distribution, the largest number of adolescents are at age 11, while the smallest group is at age 15. However, the difference between these groups is relatively minor, indicating a fairly even distribution across ages. These patterns suggest that the analytical sample is broadly representative of the adolescent population in Chinese society.

The descriptive statistics for the other key variables are presented in Table 1.

The descriptive results reveal two noteworthy initial findings. First, adolescent depression emerges as a prominent concern. The mean CES-D score exceeds 30, indicating that a substantial proportion of adolescents may be experiencing clinically relevant depressive symptoms. Second, shadow education participation varies widely across adolescents. While the average is approximately 3 h per week, the standard deviation is about 8 h, and the maximum value is as high as 100 h. This dispersion indicates that adolescents and their families make markedly different choices regarding shadow education participation, potentially leading to divergent outcomes.

### 3.3. Analytical Approach

Data analysis was conducted in four steps.

The first step involved a descriptive analysis to examine provides a descriptive overview of the annual changes in adolescent depression and shadow education in China.

The second step established a multi-level linear regression model encompassing individual, family, and community levels to examine how the duration of shadow education, family income, and survey year influence the level of depression among adolescents. This analysis aimed to evaluate whether the duration of shadow education exerts a nonlinear effect on adolescent mental health, as well as assess whether higher family income and the implementation of the “Double Reduction” policy contribute to improved mental health outcomes for adolescents.

In the third step, shadow education was categorized into three types: insufficient education, appropriate education, and excessive education. A multi-level binary logistic regression model, incorporating individual, family, and community levels, was employed to examine the impact of family income and survey year on these categories of shadow education. This analysis sought to determine tests whether higher family income and the implementation of the “Double Reduction” policy can help adolescents and their families make more reasonable decisions regarding the duration of shadow education encourage more judicious choices about shadow-education duration.

The fourth step adopted implemented three approaches to enhance robustness. First, an inverse probability weighted regression adjustment (IPWRA) model was used to control for adjusts for the confounding effects of various variables on the relationships among shadow education duration, family income, and survey year. Second, a multi-level multilevel linear regression analysis, incorporating individual, family, and community levels, was performed on subsamples with different levels of participation in shadow education to account for potential interference caused by bias introduced by COVID-19. Third, a series of sensitivity analyses based on Ordinary Least Squares (OLS) models was conducted to examine whether evaluate whether the estimated effects of shadow education duration on mental health are robust to potential biases from unobserved confounders.

All data analyses were performed using STATA software 18.0.

## 4. Results

### 4.1. Annual Changes in Depression Levels and Duration of Shadow Education

**Figure 2:**
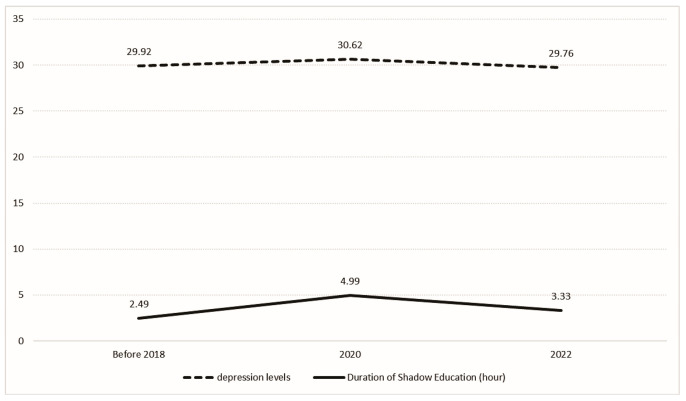
Annual changes in depression levels and duration of shadow education.

We organized the data by survey year to calculate the mean levels of depression and the average duration of shadow education among adolescents. The annual trends are displayed in Figure 2.

Figure 2 shows that both depression levels and the duration of shadow education exhibited an initial increase followed by a subsequent decrease. Prior to 2018, both indicators remained relatively low. In 2020, there was a marked uptick in both the duration of shadow education and depression levels. By 2022, however, both measures experienced a significant decline.

Three preliminary observations emerge from these patterns. Three preliminary findings emerge from these results. First, fluctuations in shadow education hours track closely with changes in depression, suggesting a potential association between the two variables. Second, the 2021 “Double Reduction” reforms may have curtailed participation, a shift that appears to coincide with the decline in depression. Third, COVID-19 likely contributed to the 2020 surge—average shadow education hours roughly doubled relative to 2018—and although the pandemic persisted into 2022, participation fell substantially, plausibly reflecting the new policy environment. Nevertheless, these descriptive patterns require formal statistical testing to establish their validity.

### 4.2. Influence of Shadow Education Duration, Family Income, and Survey Year on Depression Levels

We next tested the study hypotheses using depression levels as the dependent variable; the results are summarized in Table 2.

Model M1 serves as a null model, excluding all independent and control variables, with the primary purpose of evaluating whether a multi-level linear regression model is appropriate for analyzing adolescent depression levels. The results show that the likelihood ratio (LR) test indicates significant differences, suggesting that the multi-level linear regression model provides a better fit for the data compared to a standard linear regression model. Furthermore, the intraclass correlation coefficient (ICC) at the family level is 0.220, while the ICC at the community level is 0.053. These findings underscore the substantial within-family and within-community homogeneity in adolescent depression levels, thus justifying the use of a multi-level linear regression approach.

Building upon the null model, Model M2 incorporates control variables, in addition to the duration of shadow education and its squared term, in order to investigate the potential nonlinear association between shadow education duration and depression levels.

Most control variables demonstrate statistically significant associations with depression levels. The results suggest that younger adolescents, males, individuals with more years of schooling, greater height, better physical health, higher academic performance, fewer school absences, those serving in leadership roles, non-left-behind children, individuals experiencing lower levels of parental conflict, and those residing in communities with lower proportions of left-behind adolescents tend to report lower levels of depression, indicating more favorable mental well-being.

By contrast, variables such as weight, internet usage, and the average academic performance of adolescents within the community do not reach statistical significance, suggesting their limited impact on depression levels. One possible explanation for the insignificance of weight is that its effect may be captured by the height variable. Additionally, the average academic performance of children within the community and the proportion of left-behind adolescents may exhibit multicollinearity, potentially obscuring each other’s effects. Moreover, given the widespread prevalence of internet use among contemporary adolescents, the effect of internet usage may be relatively uniform, contributing to minimal observed variation.

**Figure 3:**
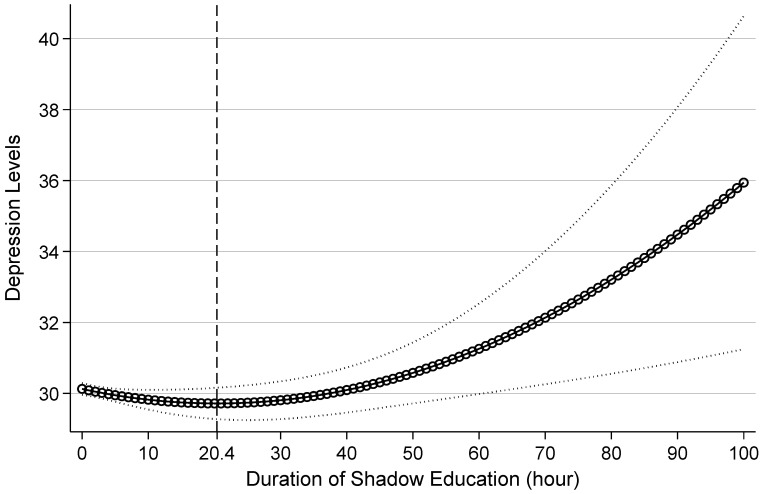
Marginal effects of shadow education duration on depression levels. The dashed line represents the confidence interval and the circle represents the marginal effect estimate.

Regarding the independent variables, the results indicate a significantly negative coefficient for shadow education duration at the 0.05 level, while the coefficient for the squared term is significantly positive at the 0.01 level. These coefficients remained relatively stable across subsequent models, suggesting a potential nonlinear relationship between shadow education duration and depression levels. To visualize this pattern, the marginal effects of shadow education duration based on Model M4 are depicted in Figure 3.

Figure 3 reveals a notable shift in the relationship between shadow education duration and adolescent depression levels at approximately 20.40 h per week. Specifically, when weekly shadow education hours range from 0 to 20.40, an increase in shadow education duration is associated with a continuous decrease in depression levels, suggesting an improvement in mental well-being. However, once the weekly duration surpasses 20.40 h, further increases are linked to rising depression levels, indicating a decline in mental health. Thus, the association between shadow education duration and adolescent depression exhibits a U-shaped pattern, while its relationship with mental health follows an inverted U-shaped curve. These results provide empirical support for Hypothesis H1.

In Model M3, family income is introduced to examine its potential association with adolescent depression. The results show that the coefficient for family income is −0.281 and statistically significant at the 0.01 level, a finding that remains consistent across subsequent models. This suggests that higher family income is associated with lower levels of adolescent depression and overall improved mental well-being. Therefore, the data supports Hypothesis H2.

**Figure 4:**
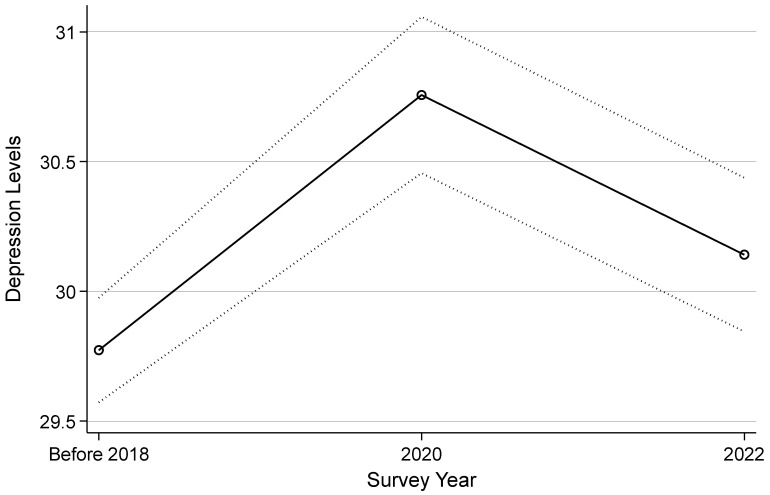
Marginal effects of survey year on depression levels. The dashed line represents the confidence interval and the circle represents the marginal effect estimate.

Model M4 further extends the analysis by incorporating the variable of survey year to assess potential changes in adolescent depression levels before and after 2021. Compared to the pre-2018 sample, the coefficients for the 2020 and 2022 samples are 0.983 and 0.368, respectively, reaching statistical significance at the 0.01 and 0.05 levels. To facilitate interpretation, a plot of the marginal effects based on Model M4 is presented in Figure 4.

Figure 4 demonstrates that the association between survey year and depression levels is consistent with the earlier descriptive statistics, exhibiting a pattern of initial increase followed by a subsequent decrease. In the samples predating 2020, adolescent depression levels exhibited a continual rise, signaling a decline in mental well-being. However, in the 2022 sample, although depression levels among adolescents remained elevated compared to those before 2018, there was a noticeable decrease compared to the 2020 sample. This suggests that, post-2021, there has been an amelioration in adolescent depression levels. Thus, the data supports Hypothesis H3.

### 4.3. Influence of Family Income and Survey Year on Shadow Education Duration

Although the hypotheses are supported, it is still necessary to clarify why adolescents from higher-income families and those surveyed in 2022 exhibit mental health advantages. One plausible explanation, as previously discussed, is that family income and the implementation of the “Double Reduction” policy may encourage families to select a more appropriate duration of shadow education, thereby promoting better mental health outcomes.

To further investigate this issue, we analyzed the influence of family income and survey timing on the selection of an optimal duration of shadow education. Prior to this analysis, it was necessary to determine a reasonable threshold for shadow education duration. In Model M4, the data-validated inflection point was found to be 20.40 h, which closely aligns with our previously estimated threshold of 21 h per week, which was based on practical experience. Therefore, it is reasonable to set 21 h per week as the upper limit for a suitable shadow education duration, as this threshold has greater practical significance.

Based on the 21 h threshold, we categorized the data into three groups: insufficient education (0 h per week), appropriate education (0.1–21 h per week), and excessive education (more than 21 h per week). Since the current version of Stata does not support multilevel regression models for unordered multinomial dependent variables, we designated “appropriate education” as the reference group and constructed two binary outcome variables: appropriate education vs. insufficient education, and appropriate education vs. excessive education. We then employed a multilevel binary logistic regression model, incorporating individual, family, and community-level factors, to examine the effects of family income and survey year on the selection of an appropriate shadow education duration. The model successfully passed the null model test, and the likelihood ratio (LR) test indicated significant differences, suggesting that the multilevel binary logistic regression model is more suitable for this analysis than a standard binary logistic regression model. The results are presented in Table 3.

Models M5 and M6 introduce the family income variable to examine how varying levels of family income are associated with the likelihood of selecting an appropriate duration of shadow education. The results from Model M5 indicate that individuals from wealthier families are 0.496 times likely to experience insufficient shadow education compared to those from less affluent backgrounds, with statistical significance at the 0.01 level. Similarly, Model M6 reveals that individuals from higher-income families are 0.774 times likely to engage in excessive shadow education than their lower-income counterparts, also significant at the 0.01 level. These findings suggest that adolescents from higher-income families are more likely to attain an appropriate duration of shadow education, supporting our earlier theoretical analysis.

Models M7 and M8 incorporate the survey year variable to assess how the selection of an appropriate shadow education duration among adolescents has changed before and after 2021. The results from Model M7 show that the odds of insufficient shadow education in the 2020 and 2022 samples are 0.648 and 0.766 times; both effects are statistically significant at the 0.01 level. In Model M8, the odds of excessive shadow education in the 2020 and 2022 samples are 1.935 times and 0.628 times, with statistical significance at the 0.01 and 0.05 levels.

These results should be interpreted in the context of the COVID-19 pandemic. The outbreak between 2018 and 2020 likely led more adolescents to participate in shadow education, an effect that persisted until 2023. Despite this increased participation during the pandemic, we did not observe a continuous rise or sustained high levels of shadow education duration. This suggests a relatively independent and distinct influence of the “Double Reduction” policy compared to the effects of the pandemic. In 2022, the likelihood of insufficient shadow education among adolescents slightly increased compared to 2020, although it remained significantly lower than in 2018. Conversely, the likelihood of excessive shadow education in 2022 significantly decreased compared to 2020. This indicates that the “Double Reduction” policy has generally guided adolescents and their families toward more reasonable decisions regarding shadow education, especially in reducing the prevalence of excessive shadow education. As a result, adolescent shadow education duration has reverted to a more appropriate range after 2021, which is consistent with our previous theoretical analysis.

### 4.4. Robustness Test

These findings should be interpreted in light of three key considerations.

First, duration of shadow education, family income, and survey timing may each be influenced by confounding factors. Because these characteristics are unevenly distributed in the population, variables such as age, gender, and years of education are simultaneously linked to participation in shadow education, family income, and survey timing, and are directly related to adolescent mental health. These variables are not randomly distributed within the population, indicating the presence of multiple confounders—such as age, gender, and years of education—which are associated with adolescents’ shadow education duration, family income, and survey timing, and also directly impact adolescents’ mental health. Although the analyses control for these factors as comprehensively as possible, residual selection bias may persist.

Second, the lasting impact of the COVID-19 pandemic must be disentangled from policy effects. Because the implementation of the “Double Reduction” reform coincided with the pandemic, distinguishing the policy signal from the pandemic signal requires multiple analytic strategies. The three-year duration of the pandemic has had widespread and enduring effects on various facets of public behavior and perceptions, which cannot be ignored. Notably, the implementation of the “Double Reduction” policy closely coincided with the pandemic, making it necessary to disentangle the effects of the policy from those of the pandemic from multiple perspectives.

Third, omitted-variable bias remains a potential threat. When relevant predictors are unobserved or unavailable in the survey, their absence can bias regression estimates if the omitted factors correlate with both the explanatory variables and the error term. In regression models, the specification of variable combinations poses a challenge to the robustness of empirical results. When necessary control variables are either unavailable in the survey data or are inherently unobservable, they cannot be included in the models. If these omitted variables are correlated with the residuals in the regression models, this may lead to biased findings. Therefore, it is important to assess the sensitivity of current findings to potential omitted-variable bias as much as possible.

To address observed confounding more rigorously, an inverse probability weighted regression adjustment (IPWRA) model was estimated to obtain average treatment effects on the treated (ATT) for shadow education duration, family income, and survey year. To address the influence of confounding variables more rigorously, we employed an inverse probability weighted regression adjustment (IPWRA) model to further examine the Average treatment effect on the treated regarding the impact of shadow education duration, family income, and survey year on adolescents’ mental health. The IPWRA procedure combines propensity score weighting with regression adjustment, delivering doubly robust estimates when either the treatment model or the outcome model is correctly specified (Cattaneo, 2010). All control variables were included in both stages, and balance diagnostics confirmed satisfactory overlap. The final results are presented in Table 4.

Because IPWRA requires categorical treatments, shadow education duration and family income were each divided into three groups. Shadow education categories matched prior analyses, whereas family income was split into tertiles.

Model M9 presents the average treatment effect on the treated (ATT) for different levels of shadow education duration on adolescent mental health. Although the coefficients cannot be directly compared with those in Model M2, a consistent nonlinear trend is observed. Compared to adolescents with insufficient shadow education, those receiving an appropriate amount exhibit significant advantages in mental health outcomes. However, adolescents with excessive shadow education do not display statistically significant benefits.

Model M10 examines the impact of different family income levels, using the low-income group as the reference. The depression levels among adolescents in the middle-income group are significantly lower compared to those in the low-income group. Additionally, adolescents in the high-income group not only exhibit substantially lower depression levels than those in the low-income group, but the absolute magnitude of the coefficient is also greater than that of the middle-income group. This finding is consistent with the results from Model M3, suggesting that higher family income may be associated with sustained improvements in adolescent mental health.

Model M11 investigates the effects of survey year, with the pre-2018 cohort serving as the reference group. Depression levels among adolescents in the 2020 group are significantly higher. Similarly, depression levels in the 2022 group are also significantly elevated compared to the pre-2018 group, although the absolute coefficient is lower than that of the 2020 group. This is in line with the results of Model M4, indicating that the implementation of the “Double Reduction” policy has contributed to relative improvements in adolescent mental health after 2021.

Overall, after accounting for a comprehensive set of confounding factors using the IPWRA model, the effects of shadow education duration, family income, and survey year remain consistent with those observed in the baseline models. This further reinforces the robustness of Hypotheses H1, H2, and H3.

To separate policy effects from pandemic effects more clearly, adolescents were stratified by shadow education participation status, and multilevel regressions were re-estimated within each stratum. This approach is based on the assumption that the impact of the COVID-19 pandemic on adolescents’ participation in shadow education and their mental health is likely broad and relatively uniform. Regardless of whether adolescents engaged in shadow education, it is probable that they experienced similar changes during the pandemic period.

In contrast, the “Double Reduction” policy specifically targets participation in shadow education, suggesting that its influence on adolescents’ mental health may differ between the two groups. Therefore, if significant differences in mental health outcomes are observed between adolescents participating in shadow education and those who do not after 2021; this would suggest that these outcomes are more likely attributable to the “Double Reduction” policy rather than to the effects of the COVID-19 pandemic.

**Figure 5:**
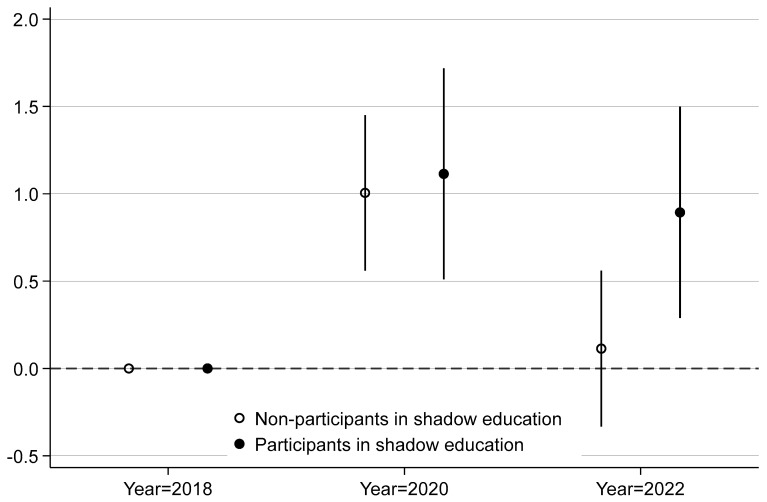
Grouped multilevel regression coefficients of survey year on depression levels (N = 7739).

To address this, we performed a stratified multilevel regression analysis based on participation status, focusing on the association between survey year and depression levels. The grouped regression coefficients are presented in Figure 5.

The results depicted in Figure 5 indicate that, using the 2018 cohort as the reference group, adolescents in the 2020 cohort exhibited a significant increase in depression levels, regardless of their participation in shadow education. This finding aligns with our theoretical analysis, suggesting that the onset of the COVID-19 pandemic had a relatively uniform and immediate negative impact on the mental health of all adolescents.

However, for adolescents in the 2022 cohort, those participating in shadow education experienced only a slight decrease in depression levels, while those not participating in shadow education showed a more pronounced decline, with no statistically significant difference compared to the 2018 cohort. This differential impact is difficult to explain solely from the perspective of the COVID-19 pandemic. Instead, the implementation of the “Double Reduction” policy may offer a plausible explanation for this phenomenon.

For adolescents engaged in shadow education, a significant proportion remain excessively involved, which considerably weakens the positive effects of the “Double Reduction” policy on their mental health. In contrast, for those not participating in shadow education, the policy has effectively reduced the social pressures and peer competition associated with shadow education. This alleviation of stress has benefited not only the mental health of these adolescents, but also the well-being of their families.

Considering both the consistency and variability in outcomes, the “Double Reduction” policy appears to exert a distinct and independent influence, separate from the effects of the COVID-19 pandemic.

**Figure 6:**
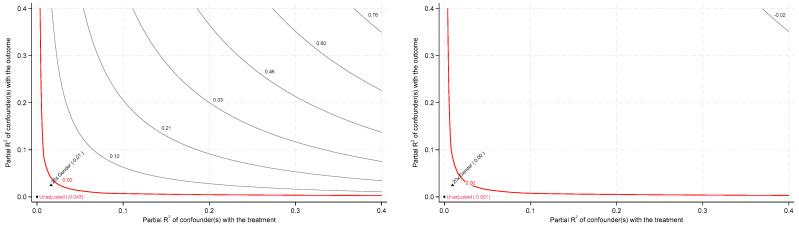
Sensitivity analysis of shadow education duration (**left**: shadow education duration; **right**: squared term of shadow education duration; N = 7739).

To further assess the sensitivity of our findings to potential omitted-variable bias, we conducted a sensitivity analysis based on the OLS model, following the approach proposed by Cinelli et al. (2020). The core idea of this analysis is to determine the degree of correlation an unobserved confounder would need to have with the key explanatory variables to invalidate the original results. If this required correlation is particularly high, it implies the greater robustness of the original findings to omitted-variable bias. The results of the sensitivity analysis are presented in Figure 6.

The results presented in Figure 6 demonstrate that, whether shadow education duration or its squared term is used as the variable of interest in the sensitivity analysis, the existing findings remain largely unchanged, even if the strength of an unobserved confounder were 20 times greater than that of the gender variable—that is, if the correlation between the unobserved confounder and either shadow education duration or its squared term were 20 times that of gender. Given that such a scenario is highly unlikely in practice, these results suggest that the estimated effects of shadow education duration are relatively insensitive to omitted-variable bias and exhibit considerable robustness.

## 5. Discussion

Based on large-scale survey data collected in China between 2016 and 2022, this study investigates the impact of shadow education duration on adolescent depression levels while considering differences arising from household income levels and policy environments. Globally, there is a growing consensus on the importance of safeguarding adolescent mental health. While the influence of education on adolescent mental health has received significant attention, most research has focused exclusively on formal schooling within educational institutions (Garaigordobil, 2023; X. Luo et al., 2022). In contrast, the increasing prevalence of shadow education worldwide has been studied primarily from the perspective of academic outcomes and social inequality (Guo et al., 2020; Jansen et al., 2023). Consequently, theoretical integration between the growing emphasis on adolescent mental health and the expanding study of shadow education remains limited. Against this backdrop, the present study seeks to bridge this gap by examining the nonlinear relationship between shadow education and adolescent mental health, while exploring the interactive influences of family and social environments. Specifically, three main theoretical findings emerge.

First, shadow education demonstrates a nonlinear effect on adolescent mental health. Using depression as the primary indicator, the duration of shadow education emerges as a critical factor in determining its psychological impact. When the duration of shadow education remains within a moderate range (0.1–20.40 h per week), increased participation appears to alleviate academic and psychological stress, supporting the view that shadow education can serve as a source of emotional support (Sun et al., 2020; Zheng et al., 2020). Within this range, shadow education contributes to improved academic performance and self-efficacy, offering psychological reassurance for both adolescents and their parents. However, when the duration exceeds this reasonable threshold (more than 20.40 h per week), further increases do not enhance mental health and may impose adverse effects. This finding aligns with recent arguments that shadow education can become a new source of psychological stress (Ahn & Yoo, 2022; Moreno & Jurado, 2024; Yao & Chen, 2023). Excessive time spent in shadow education encroaches on adolescents’ sleep and leisure time, while imposing additional academic expectations and pressures—factors that are well established as risks for deteriorating mental health (Pearlin, 1989; Xu et al., 2024).

These findings suggest that the relationship between shadow education and adolescent mental health is best characterized by a nonlinear U-shaped curve, as opposed to a simple linear association. By considering the duration of shadow education, this study offers a unified framework to address the ongoing debate: shadow education within a moderate threshold may mitigate psychological stress, whereas excessive participation imposes a new psychological burden. Notably, this type of nonlinear—U-shaped—relationship has been observed not only between shadow education and academic achievement (Hof, 2014), but also across various lifestyle factors and adolescent mental health outcomes (Qu et al., 2025). Therefore, research on shadow education or adolescent mental health should move beyond dichotomous measures (such as participation versus non-participation) and instead distinguish among insufficient, appropriate, and excessive involvement, thereby providing a more nuanced and comprehensive understanding of these trends.

Building on the foregoing discussion of potential confounders, it is equally important to recognize the methodological constraints that limit any strict causal interpretation of our results. Because the survey data do not permit full adjustment for unobservable factors—such as students’ intrinsic learning motivation, baseline academic ability, parental expectations, or classroom teaching styles (L. C. Wang, 2015)—the nonlinear association we document between shadow education hours and depressive symptoms should be understood as a descriptive pattern that emerges from the joint influence of individual, family, and institutional dynamics rather than as evidence of a deterministic causal process. In this study, the term “mental health costs” therefore denotes the statistically significant relationships detected between shadow education duration and adolescent depressive symptoms. It does not imply a proven causal pathway. Clarifying such pathways will require future research that deploys longitudinal or experimental designs capable of tracking adolescents over time and isolating time-ordered effects.

For the same reason, the estimated threshold of approximately 21 h per week should not be treated as a definitive causal benchmark. This threshold is best understood as a reference point rather than a strict boundary, recognizing the inherent variability in adolescents’ educational experiences and the potential for heterogeneity across different groups.

To probe the robustness of this inflection point, we re-specified the “appropriate education” range as 0.1–14 h (about 2 h per day) and 0.1–28 h (about 4 h per day), and then re-estimated multilevel binary logistic models using 14-hour and 28-hour cut-offs, respectively. By comparing findings across multiple thresholds, the consistency and reliability of the 21-hour benchmark could be more thoroughly assessed. The 14-hour specification yielded coefficients and levels of statistical significance that closely mirror those of the 21-hour model. Higher household income consistently reduced the odds of both insufficient education and excessive education, and the likelihood of either form of education was significantly lower in 2022 than in the pre-2018 reference period. These results indicate a stable pattern: regardless of whether the threshold is set at 14 or 21 h, higher household income remains a protective factor against both insufficient and excessive shadow education, and the prevalence of these phenomena declined following education policy shifts in 2022. These results indicate that key predictors, such as household income and survey year, remain stable across alternative threshold definitions. This consistency lends credibility to the 21-hour inflection point as a reasonable reference derived from the statistical patterns present in the data.

When the threshold was raised to 28 h, point estimates changed only marginally. Higher-income families remained significantly less likely to fall into the insufficient education and excessive education, although the latter effect was no longer statistically significant. At the 28-hour threshold, higher-income families continued to exhibit a reduced likelihood of both insufficient and excessive education; however, the effect on excessive education, while directionally similar, no longer reached statistical significance. Likewise, the 2022 survey wave was associated with significantly less insufficient education, whereas its relation to excessive education dropped below conventional significance levels. These shifts in statistical significance, particularly for excessive education, likely reflect limitations in statistical power due to a sharply reduced number of adolescents reporting more than 28 h per week of shadow education. As the cutoff increases, the pool of cases classified as excessive education diminishes, making it more difficult to detect robust associations and potentially introducing estimation bias.

To address the concern about the stability of the 21-hour threshold across subsamples, further subgroup analyses were conducted. The results are presented in Table 5.

The results reveal noticeable variations in the inflection points across these subgroups, as identified in models M12 through M17. Specifically, model M12 and M13 demonstrated that the turning point for male adolescents was approximately 18.5 h per week, while for females it was around 28 h per week. Similarly, model M14 and M15 showed that, by age group, adolescents aged 13 years and above exhibited an inflection point at 16.5 h per week, whereas those aged 12 years or below exhibited a turning point at 28.5 h per week. Finally, models M16 and M17 revealed regional differences, with the turning point for adolescents in eastern areas being approximately 25 h per week, whereas for those in central and western areas, it was significantly lower at 9 h per week.

These findings suggest that the 21-hour threshold is an aggregate average across the sample, rather than a universal standard applicable to all subgroups. The observed differences likely reflect variations in individual characteristics, cultural expectations, educational resources, and social environments, underscoring the need for nuanced interpretations of the threshold across diverse contexts.

While definitive explanations for these differences require further investigation, some plausible factors may help contextualize these findings. For instance, male adolescents might exhibit a lower inflection point compared to females due to potential differences in activity patterns and social expectations. Males may engage more frequently in outdoor activities, sports participation, or other physical endeavors that compete with shadow education time, potentially leading to earlier psychological or emotional fatigue. Additionally, family expectations and pressures may vary by gender, potentially influencing boys’ ability to balance shadow education with other obligations. However, these observations are speculative and should be validated by future research.

Similarly, the lower inflection point observed among adolescents aged 13 years or above might be linked to the increased academic intensity at the middle-school level, which often compresses the time available for shadow education. As academic demands rise, adolescents may face greater time constraints and heightened pressures, reducing the optimal duration of shadow education. Conversely, younger adolescents aged 12 years or below may experience a higher inflection point, possibly due to relatively lower academic workloads and greater flexibility in discretionary time use. These age-related differences, however, warrant further empirical exploration to confirm their validity and underlying mechanisms.

Regional differences further illustrate the importance of contextual factors. The higher inflection point in eastern areas may reflect differences in educational resources and competition levels, as families in these regions often invest more heavily in shadow education and emphasize academic achievement. In contrast, the substantially lower inflection point in central and western areas might be indicative of structural and socioeconomic disparities that constrain adolescents’ ability to engage in shadow education for prolonged periods. While these regional patterns provide initial insights, further studies are needed to understand how resource availability, cultural expectations, and local education systems interact to shape optimal participation thresholds.

These findings underscore the necessity of considering individual and contextual factors when interpreting this threshold, as applying it uniformly across diverse populations may overlook important nuances. Future research should prioritize examining these subgroup differences by using advanced methodologies to better capture the interplay between individual characteristics and social contexts in shaping the optimal duration of shadow education.

Taken together, these robustness checks suggest that the overall direction of income and cohort effects is stable across alternative definitions of ‘appropriate’ shadow education duration, yet they also underscore the provisional nature of the 21-hour threshold. While this figure is supported by statistical regularities and subgroup analyses, it should still be regarded as a practice-informed approximation rather than a definitive causal boundary. The variability in inflection points across subsamples highlights the need to account for heterogeneity when interpreting the threshold and applying it to different contexts. That figure arises from practice-informed observation and from the statistical regularities of the present dataset. It should be regarded as an initial reference point rather than a hard causal cut-off. Identifying optimal thresholds—and understanding the conditions under which shadow education becomes harmful—requires more rigorous causal inference strategies that explicitly model heterogeneity across students, families, schools, and regions. Longitudinal panel studies or randomized interventions would be particularly valuable for determining whether similar thresholds emerge in other contexts and for explicating the mechanisms through which shadow education intensity translates into mental-health outcomes.

Second, adolescents from high-income families are more likely to avoid the detrimental effects of shadow education on mental health. As a component of overall well-being, adolescent mental health is also influenced by a range of family and social factors, consistent with the theory of the social determinants of health (Nutbeam & Lloyd, 2021; Yeung, 2021). However, there is ongoing debate regarding the influence of family income on education and mental health. Some perspectives emphasize that high-income families may hold stronger educational expectations and set higher academic goals for their children, often adopting a strategy of concerted cultivation. When adolescents fail to meet these expectations, the resulting gap may intensify psychological stress and mental health problems (L. Zhang, 2024). Despite these considerations, the findings of this study predominantly support the positive effects of family income. Specifically, adolescents from higher-income families not only have more opportunities to participate in shadow education, but are also more likely to select an appropriate duration of shadow education with parental support. Abundant family resources help adolescents in high-income families to better avoid the psychological stress associated with excessive shadow education and to develop stronger coping mechanisms and channels for stress relief (Bray, 2023; Song & Zhou, 2019).

Why, then, does higher family income tend to yield more positive mental health outcomes in the context of shadow education? One plausible explanation is that the ability of high-income families to provide both appropriate levels of shadow education and more effective channels for stress relief plays a more significant role in mediating the impact of shadow education on adolescent mental health, effectively offsetting the negative influences of higher parental expectations. Of course, whether this is the true underlying mechanism still requires more rigorous empirical testing and theoretical validation.

Extending from this, the study highlights how the negative mental health effects of shadow education are unevenly distributed across different groups. The discussion around shadow education often emphasizes its role in promoting academic achievement, and debates whether such academic consequences challenge educational equity and social equality (Jansen et al., 2023; W. Zhang & Bray, 2018). However, as a mechanism for the reproduction of social inequality, the connection between shadow education and social inequality is not limited to academic performance; it also encompasses mental health. Adolescents from disadvantaged backgrounds face greater challenges in gaining academic benefits from suitable shadow education and are more likely to bear the mental health costs caused by both insufficient and excessive education. This suggests that the impact of shadow education on social inequality is multifaceted: it not only involves the allocation of educational resources and improvement of academic achievement, but also extends deeply into the subjective realm of adolescents by shaping inequalities in mental health.

Third, the relationship between shadow education and adolescent mental health also reflects the influence of policy transformation. The implementation of the “Double Reduction” policy in 2021 marked a significant turning point in China’s regulatory framework for shadow education, shifting from a laissez-faire model to a more regulated approach (W. Zhang, 2023, p. 61). This policy transition has substantial implications for adolescents’ participation in shadow education and its effects on mental health.

However, it is important to recognize the potential confounding impact of the COVID-19 pandemic. Both the pandemic itself and related containment policies significantly increased the demand for shadow education outside schools, while posing enduring challenges to the mental health of adolescents and their families (Du, 2024; Houghton et al., 2022). In this context, the “Double Reduction” policy has produced opposing trends. Following its introduction in 2021, the overall duration of adolescents’ shadow education declined, while indicators of mental health, such as depression levels, improved. This suggests that, under stricter regulation, the public may adopt a more rational and pragmatic approach to shadow education, placing greater emphasis on ensuring appropriate participation for adolescents. Another important factor may be that the “Double Reduction” policy has, to some extent, weakened the legitimacy of shadow education, thereby reducing the peer pressure associated with participation—a factor that often drives irrational involvement (Kim et al., 2022; Pan et al., 2022).

By examining the impact of the “Double Reduction” policy, this study incorporates the influence of educational regulatory policies and their changes into the analysis of shadow education. Most extant research on shadow education is conducted within the context of a single country, often overlooking the pivotal role of regulatory frameworks (Hertz et al., 2022; Mikkonen et al., 2020). The major regulatory shift brought by China in 2021 offers an ideal case for investigating this dimension. It suggests that differences in regulatory frameworks across countries may systematically shape adolescents’ engagement in shadow education and their mental health outcomes. The implications of this finding extend beyond China, as the regulation of shadow education is a challenge faced by all nations and constitutes a critical component of each country’s educational ecosystem (J. Luo & Chan, 2022). Specifically, the “Double Reduction” policy aims to minimize the negative effects of excessive shadow education by imposing restrictions on after-school tutoring and reducing academic burdens, thereby contributing to improved student well-being.

China’s “Double Reduction” policy serves as a unique regulatory experiment, offering insights that can inform global approaches to managing the challenges posed by shadow education. Unlike the laissez-faire approach prevalent in countries such as the United States or Canada—where private tutoring is largely unregulated and market-driven—China’s policy represents a strong, state-led intervention. This comparative perspective highlights how regulatory frameworks profoundly shape not only the scale of shadow education, but also its broader social and psychological consequences. One of the key mechanisms of the “Double Reduction” policy is the direct restriction of commercial tutoring services. Through imposing strict limits on private tutoring institutions, especially those focused on core subjects, and converting for-profit organizations into non-profits, the policy seeks to curtail the excessive expansion of the shadow education industry. This is intended to alleviate students’ academic pressure and time burden, affording them greater opportunities for rest, extracurricular activities, and holistic development. Furthermore, the policy tasks schools with greater responsibility for student learning and well-being, mandating enhanced after-school programs, diversified enrichment activities, and targeted academic support within the public education system. This school-centered approach not only reduces dependence on the private market, but also promotes educational equity by ensuring all students—regardless of socioeconomic background—have access to additional resources and support. Internationally, China’s experience demonstrates that robust policy interventions can effectively rebalance the relationship between formal and shadow education and mitigate the negative outcomes associated with unregulated private tutoring. For countries grappling with the rapid expansion of shadow education, the “Double Reduction” policy highlights the necessity of coordinated action by governments, schools, and society at large. It also raises important questions about how to develop policies that are contextually appropriate, achieve a balance between academic competitiveness and student well-being, and avoid the unintended consequences of both over-regulation and unchecked market freedom.

Returning to the Chinese context, it is clear that the impact of the “Double Reduction” policy warrants further exploration. A comprehensive understanding of this policy’s consequences requires attention not only to its immediate educational outcomes, but also to the broader social and cultural environment in which it has been implemented. This includes the need for more rigorous causal inference strategies to disentangle its effects from those of the COVID-19 pandemic, as well as placing educational regulatory policies and their transformations within broader socio-cultural dynamics. For example, it remains to be studied whether the impact of the “Double Reduction” policy differs systematically between urban and rural areas—specifically, whether it exacerbates or alleviates educational inequality across these contexts. Urban–rural disparities represent a critical dimension for analysis: urban students often benefit from greater access to alternative educational resources and a wider array of non-academic enrichment activities, whereas rural students may face ongoing structural limitations, such as fewer extracurricular options and less institutional support, potentially magnifying pre-existing inequalities. Urban students may have greater access to alternative educational resources and non-academic enrichment activities, while rural students might face persistent structural limitations, potentially leading to uneven policy effects. Furthermore, the role of parental expectations, which are often closely tied to socioeconomic status, merits careful consideration (L. C. Wang, 2015). Families from higher income strata may seek alternative forms of support or extracurricular activities to compensate for the reduction in formal shadow education, potentially maintaining or even widening existing educational disparities. In contrast, lower-income families might lack the resources to pursue such alternatives, thus experiencing the policy’s effects differently. Additionally, parental expectations—often shaped by socioeconomic status—play a pivotal role in mediating the policy’s impact (L. C. Wang, 2015; Yeung, 2024). Families from a higher-income strata may compensate for reduced opportunities in formal shadow education by investing in alternative forms of academic or extracurricular support, thereby maintaining or even accentuating pre-existing educational advantages. Conversely, lower-income families may lack the financial or informational resources to access such alternatives, resulting in a differentiated experience of the policy’s consequences. Moreover, adolescents’ access to non-academic enrichment opportunities—such as sports, arts, and community engagement—varies greatly by region and family background, and these opportunities can play a critical role in shaping psychological well-being and holistic development post policy intervention. The availability and uptake of non-academic enrichment, including sports, arts, and civic engagement, are highly uneven across both geographic and socioeconomic lines; these experiences are increasingly recognized as key for adolescent psychological well-being, resilience, and holistic development in the context of reduced shadow education. Understanding how the “Double Reduction” policy interacts with these sociocultural variables is essential for evaluating its long-term impacts and for informing more equitable policy adjustments to address the diverse needs of Chinese adolescents and their families. A nuanced understanding of how the “Double Reduction” policy interacts with such sociocultural variables is essential for a comprehensive evaluation of its long-term impacts. Careful attention to these dynamics will be crucial for informing the ongoing design and refinement of educational policies that not only enhance equity, but also support the diverse developmental and psychosocial needs of Chinese adolescents and their families.

This research carries several social implications and provides valuable directions for future development. Firstly, how should the goals of shadow education regulatory policies be defined? The formulation of educational policies is essential for societal development, yet it often involves contradictions and debates (L. Wang & Hamid, 2024). Based on the findings of this study, the objective of regulation should not be to eliminate shadow education entirely, but rather to maintain it at a reasonable level of intensity and duration. Such an approach would allow for shadow education to effectively enhance adolescents’ academic performance while minimizing excessive mental health costs. This highlights the importance of determining an optimal duration for shadow education, which may vary across different cultural and social contexts. Future research should prioritize comparative studies that examine the mental health consequences of shadow education across various countries, thereby providing insights into how cultural differences shape its impacts.

Secondly, beyond participation in shadow education, how adolescents engage with it and the types of shadow education they choose to pursue are also critical issues worth considering. The duration of shadow education is not merely a key factor influencing mental health; it reflects certain characteristics and dimensions of shadow education, constituting a broader and more complex system. The implications of this study suggest that shadow education is not a homogeneous entity, but rather encompasses a variety of differences. The manner in which adolescents engage in shadow education requires further exploration. For instance, whether shadow education is conducted through one-on-one sessions or in group settings, whether shadow educators undergo professional training, and whether the content of shadow education aligns with formal school curricula are all key characteristics that may influence the outcomes (Hertz et al., 2022). Particularly in the era of widespread digital technology, shadow education has taken on more flexible forms (Bray, 2024). It remains to be determined whether online and offline forms of shadow education led to differing academic performance and mental health outcomes. These endeavors not only necessitate a more systematic delineation of the theoretical underpinnings of shadow education, but also require updated methods of data collection to obtain more targeted survey data.

Finally, the unequal characteristics of shadow education and their implications for mental health demand further investigation. Shadow education, as an avenue for transmitting and accumulating economic, social, and cultural capital, is deeply embedded within wider societal structures of inequality, and its mental health consequences cannot be understood in isolation from these broader contexts (Yeung, 2022). As a form of economic capital, family income plays a critical role in shaping the mental health consequences of shadow education, highlighting its intersection with broader mechanisms of social inequality. These inequalities can manifest at multiple levels, including familial, urban–rural, and regional disparities. For instance, at the familial level, a family’s social resources often encompass multiple dimensions—such as economic, social, and cultural capital—which interact and transform one another. Different combinations of these capitals may result in varying mental health outcomes associated with shadow education. At the urban-rural level, disparities in educational resource distribution further exacerbate inequalities. Adolescents in resource-rich urban areas benefit from higher-quality formal education and shadow education, gaining greater access to prestigious universities and adopting more rational attitudes toward shadow education. In contrast, adolescents in resource-scarce rural areas face dual disadvantages from both formal and shadow education, experiencing higher mental health costs. At the regional level, apart from differences in educational resources, cultural and conceptual factors are also highly significant. Specifically, different regions of China may maintain distinct parenting cultures and values, which not only affect the types and extent of shadow education parents are willing to invest in, but also shape parent–adolescent emotional interaction patterns. As a result, the relationship between adolescent shadow education and mental health may systematically differ across regions, shaped jointly by regional resources and cultures. Future research would benefit from examining how specific configurations of family capital intersect with contextual factors, such as regional educational competition and urban–rural disparities, to produce differentiated mental health outcomes. These investigations would provide deeper insights into the complex dynamics of shadow education and its broader social implications.

In addition to the aforementioned structural and contextual disparities, it is essential to account for several factors that may confound the observed associations between shadow education and adolescent mental health. Academic motivation, for example, can significantly influence both the likelihood of participating in shadow education and the ways in which such participation impacts psychological well-being. Highly motivated students may self-select into shadow education environments, potentially deriving positive or negative mental health effects depending on their coping strategies and achievement orientations (Kapadia, 2024). Similarly, adolescents’ baseline mental health conditions represent a critical source of heterogeneity: those with pre-existing vulnerabilities may respond differently to the demands of shadow education than their peers with greater psychological resilience (Bray, 2014), thereby complicating any straightforward interpretation of mental health outcomes. Furthermore, school climate—including features such as peer support, teacher–student relationships, and institutional emphasis on holistic development—may either buffer or exacerbate the psychological effects of shadow education (Podiya et al., 2025). These factors could interact with both family and regional characteristics, ultimately shaping the relationship between shadow education and adolescent mental health in complex and context-dependent ways. Future research should employ designs that explicitly control for or account for these potential confounders, which would greatly strengthen the credibility and interpretability of findings in this domain.

## 6. Limitations

This study has several limitations that should be acknowledged. Firstly, the influence of endogeneity must be approached with caution. The primary findings of this research are based on regression analyses, where the relationships among variables can only be explored at a correlational level, making it challenging to establish strict causal relationships. This choice was primarily made because one of the core objectives of the study is to reveal the nonlinear relationship between the duration of shadow education and adolescents’ mental health. To achieve this, incorporating the duration of shadow education and its quadratic term into the regression model is a commonly used method. However, corresponding causal inference techniques are still relatively underdeveloped, making it difficult to fully meet research needs. While the impact of non-random errors exists, it is unlikely to fundamentally alter the results, especially given the robustness checks conducted. After categorizing the variables and controlling for observable confounding effects using the IPWRA model, the results remained entirely consistent with those obtained from the regression model. Sensitivity analysis based on OLS further confirmed that the existing findings demonstrate a certain degree of robustness even in the presence of potential unobservable omitted variables. This further strengthens our confidence in the conclusions drawn from the data. However, caution is still warranted in extrapolating the results, as IPWRA can only control for observable confounding effects, and unobservable confounding influences may still exist. For instance, the impact of the “ability” variable on the relationships between shadow education, academic performance, and mental health warrants particular attention. To address these potential endogeneity issues, more rigorous causal inference techniques will be necessary.

Secondly, separating the effects of the “Double Reduction” policy and the COVID-19 pandemic requires further effort. Both occurred within the 2018–2022 timeframe and significantly influenced shadow education and mental health during this period, presenting the greatest challenge in examining the impact of the “Double Reduction” policy through survey data. This study approaches this issue from two angles: first, it posits that the effects of the COVID-19 pandemic and the “Double Reduction” policy on shadow education and adolescents’ mental health are inconsistent. Given that the pandemic led to an overall increase in the duration of shadow education and a decline in adolescents’ mental health, any data that contradicts this trend is likely attributable to the relatively independent effects of the “Double Reduction” policy. Second, it suggests that the characteristics of the impacts of the COVID-19 pandemic and the “Double Reduction” policy on shadow education and adolescents’ mental health are also inconsistent. The pandemic may have a more uniform effect on adolescents participating in shadow education and those not participating, while the “Double Reduction” policy has a more direct association with shadow education, potentially leading to more differentiated impacts across varying levels of participation. This is supported by the data. However, this attempt is far from perfect, as the underlying assumptions are quite strict and may not fully align with social realities. Given that both the “Double Reduction” policy and the COVID-19 pandemic are significant social events with widespread impacts during this period, their influences not only overlap in time, but may also be interrelated, exhibiting both generality and variability. Therefore, it is crucial to investigate trends in shadow education duration and depression levels over a longer timeframe. Extending the research period would allow for a better identification of the effects of different stages of the COVID-19 pandemic on shadow education and mental health, which is key to disentangling the two influences. It would also facilitate a more systematic presentation of the long-term effects of regulatory changes in China’s shadow education landscape. Additionally, incorporating questions and variables related to the COVID-19 pandemic into social surveys and research would help better control for and separate its effects.

Lastly, in-depth qualitative research is essential for enhancing the systematic understanding of the relationship between shadow education and mental health. Certain data phenomena and research questions remain challenging to address through quantitative analysis alone. For instance, why are individuals with lower body weights more likely to be included in the missing data? Why does the average academic performance of adolescents at the community level fail to exert a significant impact on their mental health? Why did the probability of insufficient shadow education among adolescents slightly increase in 2022 compared to 2020? How do different families determine what constitutes a reasonable duration for shadow education? These phenomena and questions highlight the limitations of quantitative methods in capturing the nuanced and contextual factors underlying these observations. To address these gaps, direct engagement with families involved in shadow education and interviews with adolescents themselves are necessary. Such qualitative approaches can provide richer, more detailed information that complements quantitative findings, offering deeper insights into the mechanisms at play. Specifically, qualitative research can help elucidate some of the puzzling data results observed in this study, such as the patterns of missing data or the lack of a significant community-level effect. Furthermore, it can enhance our understanding of how families navigate decisions regarding shadow education duration and its implications for adolescents’ mental health.

## 7. Conclusions

Drawing on four large-scale surveys conducted in China between 2016 and 2022, this study investigates the mental health implications of shadow education. Specifically, it examines how the duration of shadow education influences adolescents’ depression levels and explores how this relationship varies across different family backgrounds and survey time points.

This study yields several key insights, while also highlighting important limitations. First, the relationship between shadow education duration and adolescent depression appears to follow a U-shaped pattern. When participation remains within a moderate range—up to approximately 20.40 h per week—shadow education is associated with lower reported levels of depression. However, when participation exceeds this threshold, further increases in duration are linked to higher depression levels. Second, adolescents from higher-income families may have access to more resources and support, enabling them to engage in shadow education in a more measured manner and potentially experience lower levels of psychological distress. Third, there is an observable trend toward more moderate shadow education participation and declining depression levels after the introduction of regulatory policies in China post-2021. However, it is important to note that these patterns are correlational and cannot be interpreted as evidence of direct causal effects, given the study’s cross-sectional nature and the absence of random assignment or longitudinal follow-up. Additionally, the potential confounding impact of the COVID-19 pandemic and other unmeasured factors may also influence both shadow education practices and adolescent mental health outcomes. Future research utilizing longitudinal or experimental designs will be necessary to more rigorously assess the causal mechanisms underlying these associations.

Overall, this study avoids the oversimplification of previous research, which often characterized the impact of shadow education on mental health as either uniformly positive or negative. Instead, it highlights shadow education duration as a critical determinant, demonstrating that the duration largely dictates whether shadow education serves as a mechanism for mitigating mental stress or as a new source of psychological pressure. Furthermore, this study incorporates the influences of family background and the “Double Reduction” policy into its analysis. It underscores that, while shadow education carries mental health costs, these costs are distributed unequally among adolescents and across society. Adolescents from higher-income families are more likely to experience lower mental health costs associated with shadow education, whereas regulatory policies and changes in shadow education practices across different contexts may reshape the relationship between shadow education and mental health.

In addressing the complex relationship between adolescent shadow education and mental health, governments, schools, and families should share corresponding responsibilities. For the government, the fundamental approach is to provide more public-oriented and high-quality education services through the formal education system. In particular, leveraging digital technology to develop open online learning platforms can offer substantial support. Establishing special funds to assist low-income families—ensuring they have access to ample formal and informal educational opportunities—is also a necessary measure to promote educational equity.

In addition, governments should consider issuing specific guidelines regarding ideal shadow education duration to mitigate the risks associated with excessive engagement. Based on current findings, policymakers could recommend a weekly maximum of 21 h of shadow education for adolescents, while also suggesting lower thresholds for younger children and tailored benchmarks for different demographics. For instance, the optimal duration of shadow education could vary based on socioeconomic status, geographical location, or cultural norms. Governments might conduct large-scale surveys to determine these recommended thresholds and integrate this data into targeted interventions. Specific programs could be designed for low-income families that focus on balanced shadow education participation, preventing overreliance on shadow education as a means of achieving academic competitiveness. These guidelines would serve as a practical tool for families and schools to better manage shadow education participation, thereby reducing its potential negative impacts on mental health and general well-being.

For schools, their role extends beyond formal education to providing structured support for students participating in shadow education. Proactively offering more flexible and targeted forms of shadow education as a supplement to formal schooling can be beneficial, provided that the content emphasizes supporting students’ holistic development rather than reinforcing rigid academic competition. For instance, shadow education programs designed by schools could prioritize skills such as critical thinking, creativity, and emotional resilience, helping students develop competencies essential for long-term success. Schools should also establish mechanisms to monitor students’ shadow education engagement levels and identify signs of potential stress or burnout.

In cases of over-engagement, schools could provide timely interventions, including counseling or mental health support tailored specifically to shadow education participants. Such programs might include workshops on stress management, mindfulness training, and peer support groups, equipping students with tools to navigate the emotional pressures associated with shadow education. Additionally, schools could collaborate with mental health professionals to offer individualized psychological counseling for students experiencing significant stress or burnout due to shadow education participation. Recognizing the particular challenges faced by students from low-income families, schools could introduce subsidized shadow education programs that emphasize balance and equity. These programs should be designed to promote academic progress without compromising mental health, addressing the unique stressors experienced by economically disadvantaged students. Furthermore, schools should incorporate physical and mental health education into their curricular design to provide foundational knowledge on managing stress and maintaining emotional well-being.

For parents, their role in managing shadow education is equally critical. Parents need to fully respect their children’s interests and preferences, avoid comparison and herd mentality, and place a strong emphasis on their children’s mental health. Governments and schools could work collaboratively to deliver parenting workshops or seminars that equip parents with evidence-based strategies for supporting adolescents’ mental health and academic development. For example, workshops could help parents recognize signs of stress or burnout in their children, understand recommended durations of shadow education, and develop effective communication strategies to support their children’s well-being. Parents should also be encouraged to foster open dialogs with their children about their academic goals and emotional needs, ensuring that shadow education aligns with their children’s interests and does not compromise their overall development.

Moreover, parents should be educated on the risks of excessive shadow education and its potential impact on mental health. This could include promoting balanced schedules that integrate sufficient rest, leisure, and social activities. Parenting programs could also provide practical tools to help families establish routines that prioritize mental health while accommodating academic aspirations. For example, governments could introduce digital platforms or mobile applications designed to help parents track and optimize their children’s shadow education hours, balancing academic goals with psychological well-being.

Taken together, this framework aims to provide a concrete and actionable approach for addressing the interplay between shadow education and adolescent mental health. By setting specific guidelines for shadow education hours, establishing targeted support programs for low-income families, and implementing tailored mental health interventions, policymakers, schools, and families can work collectively to mitigate risks and promote the well-being of students participating in shadow education.

## Figures and Tables

**Table 1:** Descriptive statistics of key variables (N = 7739).

Variable	Mean	SD	Min	Max
Level of depression	30.068	6.407	20	72
Duration of shadow education (hour)	3.212	8.231	0	100
Age (year)	12.378	1.678	10	15
Gender	0.535	0.499	0	1 = Male
Years of education (year)	6.354	1.827	1	12
Height (cm)	152.479	13.886	50	190
Weight (Jin ≈ 0.5 kg)	85.937	24.191	30	190
Physical health	4.053	0.905	1	5 = very healthy
Academic performance (%)	30.836	22.057	5	88
Absenteeism	0.013	0.113	0	1 = has absenteeism
Class leadership	0.335	0.472	0	1 = holds class leadership
Internet usage	0.630	0.483	0	1 = uses the Internet
Left-behind child	0.535	0.499	0	1 = not a left-behind child
Arguments with parents	1.209	2.977	0	50
Community children’s mean academic performance (%)	30.876	9.329	5	88
Community mean percentage of non-left-behind children	62.876	20.691	0	100

**Table 2:** Multi-level linear regression model for depression levels (N = 7739).

	M1	M2	M3	M4
Variables	Depression Levels	Depression Levels	Depression Levels	Depression Levels
Control variable	NO	YES	YES	YES
Duration of Shadow Education		−0.042 **	−0.036 *	−0.040 **
		(0.019)	(0.019)	(0.019)
Duration of Shadow Education Square Item		0.001 ***	0.001 **	0.001 **
		(0.000)	(0.000)	(0.000)
Family Income			−0.281 ***	−0.301 ***
			(0.082)	(0.082)
Before 2018 (base group)				
2020				0.983 ***
				(0.180)
2022				0.368 **
				(0.182)
Constant	30.030 ***	32.661 ***	34.984 ***	35.817 ***
ICC of Family Level	0.220	0.189	0.187	0.187
ICC of Community Level	0.053	0.065	0.066	0.074

Notes: 1. Standard errors in parentheses 2. *** *p* < 0.01, ** *p* < 0.05, * *p* < 0.1. 3. ICC means intraclass correlation coefficient.

**Table 3:** Multilevel binary logistic regression model of shadow education duration (using appropriate education as the reference category).

	M5	M6	M7	M8
Variables	Insufficient Education	Excessive Education	Insufficient Education	Excessive Education
Control variable	YES	YES	YES	YES
Family income	0.496 ***	0.774 ***	0.502 ***	0.785 ***
	(0.024)	(0.063)	(0.025)	(0.066)
Before 2018 (base group)				
2020			0.648 ***	1.935 ***
			(0.057)	(0.335)
2022			0.766 ***	0.628 **
			(0.067)	(0.126)
Constant	19,344.301 ***	0.476	12,299.165 ***	0.464
	(13,851.352)	(0.669)	(8946.199)	(0.671)
ICC of family level	0.564	0.492	0.573	0.526
ICC of community level	0.101	0.466	0.120	0.526
Observations	7439	2424	7439	2424

Notes: 1. Coefficients represent odds ratios; standard errors in parentheses 2. *** *p* < 0.01, ** *p* < 0.05.

**Table 4:** Inverse probability weighted regression adjustment model for depression levels (N = 7739).

	M9	M10	M11
Variables	Depression Levels	Depression Levels	Depression Levels
Control variable	YES	YES	YES
Insufficient education (base group)		YES	YES
Appropriate education	−0.378 **		
	(0.184)		
Excessive education	0.012		
	(0.384)		
Low family income (base group)	YES		YES
Medium family income		−0.505 ***	
		(0.184)	
High family income		−0.603 ***	
		(0.199)	
Before 2018 (base group)	YES	YES	
2020			1.143 ***
			(0.209)
2022			0.717 ***
			(0.219)

Notes: 1. Robust standard errors in parentheses 2. *** *p* < 0.01, ** *p* < 0.05.

**Table 5:** Multi-level linear regression model for depression levels.

	M12 (Male)	M13 (Female)	M14 (Above 13 Years Old)	M15 (Below 12 Years Old)	M16 (Eastern)	M17 (Central and Western)
Variables	Depression Levels	Depression Levels	Depression Levels	Depression Levels	Depression Levels	Depression Levels
Control variable	YES	YES	YES	YES	YES	YES
Duration of shadow education	−0.037	−0.056 **	−0.033	−0.057 **	−0.100 ***	−0.018
	(0.025)	(0.028)	(0.029)	(0.024)	(0.032)	(0.023)
Duration of shadow education square item	0.001 **	0.001 *	0.001	0.001 **	0.002 ***	0.001
	(0.001)	(0.001)	(0.001)	(0.001)	(0.001)	(0.001)
Constant	33.627 ***	30.598 ***	33.517 ***	32.479 ***	33.032 ***	32.481 ***
	(1.289)	(1.659)	(2.423)	(1.575)	(2.252)	(1.150)
Observations	4137	3602	3630	4109	1943	5796

Notes: 1. Standard errors in parentheses. 2. *** *p* < 0.01, ** *p* < 0.05, * *p* < 0.1. 3. ICC omitted.

## Data Availability

Readers can access the data used in the study from https://cfpsdata.pku.edu.cn (accessed on 20 June 2025).
